# Neurotechnology-enabled closed-loop optogenetics for modulating autoimmune glial responses in experimental ADEM

**DOI:** 10.1097/MS9.0000000000005093

**Published:** 2026-05-06

**Authors:** Ayesha Tariq, Malaika Khalid, Eman Fatima, Zehra Batool, Mahnoor Fatima

**Affiliations:** a Department of Medicine, Punjab Medical College, Faisalabad, Pakistan; b Department of Medicine, King Edward Medical University, Lahore, Pakistan; c University Institute of Physical Therapy, University of Lahore, Lahore, Pakistan; d Department of Medicine, Bahria Medical University of Health Sciences, Karachi, Pakistan; e Department of Medicine, Shaheed Ziaur Rahman Medical College, Rajshahi Medical University, Bogura, Bangladesh

*To the Editor*,

Acute disseminated encephalomyelitis (ADEM) is an immune-mediated, inflammatory demyelinating disease of the central nervous system with an incidence of 0.4–0.8 per 100 000 children per year and peak occurrence in those younger than 10 years. Most patients recover clinically within 3 months, whereas relapse occurs in 20–30% of cases. It typically presents as monophasic with multifocal white matter involvement, but multiphasic forms may also occur. Pathologically, it includes perivenous inflammation, blood–brain barrier alteration, and immune cell infiltration. These clinical features emphasize the need for targeted and mechanism-specific therapeutic strategies^[^1^]^. Optogenetics includes integrated optics and genetic methods that help in the precise stimulation of astrocytes via the expression of light-sensitive channels and receptors. It thus targets the specific brain area to regulate its activities. Development of such novel approaches might be helpful to monitor and control the functions of astrocytes^[^2^]^.

Experimental studies show that real-time and continuous closed-loop control of brain activity can be achieved by pairing the electrical recordings and optogenetics. The closed-loop optogenetic stimulation with excitatory opsins enables the precise manipulation of neural dynamics in brain cells^[^3^]^. Therefore, we hypothesize that neurotechnology-enabled closed-loop optogenetics can be used for modulating autoimmune glial responses in experimental ADEM.

Neurodegenerative diseases are progressive disorders characterized by synaptic loss and neuronal death. Optogenetics involves the use of genetic and optical methods to control the activity of specific cell types. The technique has revolutionized the field of neuroscience by enabling precise control of neural activity through light-sensitive proteins known as opsins^[^2,4^]^.

Evidence studies have shown that optogenetics has been used to regulate astrocyte activity, which in turn maintains blood–brain barrier integrity^[^5^]^. In ADEM, there is an increased permeability of the blood–brain barrier due to reactive astrocytes. Therefore, the efficacy of this approach can be utilized for the therapeutic intervention of the disease. More recent studies show that reactive astrocytes also play a crucial role in demyelination and remyelination of MS^[^6^]^. Demyelination occurs in ADEM, so by using optogenetics, astrocytes can also cause remyelination in ADEM.

Incorporating optogenetics into the study of neurodegenerative diseases like AD has resulted in a significant leap in this field during the last few decades, kicking off a revolution in our knowledge of the networks that underpin cognitive functions^[^7^]^. However, research studies have yet to demonstrate its direct application as a therapeutic modality in acute demyelinating conditions such as ADEM. Current research indicates that optogenetic methods can affect glial and neuronal activity. They are also being investigated for neural tissue regeneration and glial phenotypic regulation, two processes that are pertinent to demyelinating illnesses. Despite that, implementing this technique in ADEM remains limited, largely due to the challenges of rapidly evolving inflammatory conditions, the invasiveness of light delivery systems, and the lack of specific disease models that would allow meaningful preclinical optogenetic intervention in ADEM-like pathology^[^8^]^.

In conclusion, neurotechnology-enabled closed-loop optogenetics enables a focused approach in controlling autoimmune glial responses in demyelinating diseases. This method allows for specific regulation of microglial activity during neuroinflammation by combining adaptive light-based modulation with real-time neurological monitoring. The integration of optogenetics into neurodegenerative disease research has significantly advanced in the field, offering new insights and paving the way for Innovative treatment strategies. Its application in clinical settings, although still in the nascent stages, suggests a promising future for addressing some of the most challenging aspects of neurodegenerative disorders

## Data Availability

Not applicable to this study.
